# Alterations in Cell-Extracellular Matrix Interactions during Progression of Cancers

**DOI:** 10.1155/2012/219196

**Published:** 2012-01-04

**Authors:** Rajeswari Jinka, Renu Kapoor, Pavana Goury Sistla, T. Avinash Raj, Gopal Pande

**Affiliations:** ^1^Department of Biochemistry, Acharya Nagarjuna University, Guntur 522510, India; ^2^Centre for Cellular and Molecular Biology, Council of Scientific and Industrial Research (CSIR), Uppal Road, Hyderabad 500 007, India

## Abstract

Cancer progression is a multistep process during which normal cells exhibit molecular changes that culminate into the highly malignant and metastatic phenotype, observed in cancerous tissues. The initiation of cell transformation is generally associated with genetic alterations in normal cells that lead to the loss of intercellular- and/or extracellular-matrix- (ECM-) mediated cell adhesion. Transformed cells undergo rapid multiplication and generate more modifications in adhesion and motility-related molecules which allow them to escape from the original site and acquire invasive characteristics. Integrins, which are multifunctional adhesion receptors, and are present, on normal as well as transformed cells, assist the cells undergoing tumor progression in creating the appropriate environment for their survival, growth, and invasion. In this paper, we have briefly discussed the role of ECM proteins and integrins during cancer progression and described some unique conditions where adhesion-related changes could induce genetic mutations in anchorage-independent tumor model systems.

## 1. Introduction

Cancer afflicts an organ or a tissue by inducing abnormal and uncontrolled division of cells that either constitute it or migrate to it. At the cellular level, this is caused by genetic alterations in networks that regulate cell division and cell death. The increased rate of proliferation of transformed cells causes further mutations in genes that regulate other cellular processes. For example, transformed cells eventually gain the capacity to invade into other tissues by modulating their own kinetic properties without losing the capacity to divide rapidly and avoid cell death, despite internal and external perturbations.

Cancer cells adopt diverse mechanisms to cope with the various physiological insults, such as low oxygen and metabolic stress, that they encounter [1]. These mechanisms have been discussed in a recent review [2], and based upon that discussion, six important hall marks of cancer cells can be identified. These are (a) sustained proliferative signalling, (b) evasion of growth suppressors, (c) resistance to cell death, (d) replicative immortality, (e) copious angiogenesis, and (f) active invasion and metastasis. In addition to these, cancer cells can exhibit two other properties, that is, tumor promoting inflammation and gene instability that assist the cells in the transition from normal to oncogenic phenotype [2]. Eventually transformed cells undergo somatic evolution and generate diverse populations that tend to harbor genetic and epigenetic instabilities and alterations [3]. These changes also assist the cells in adapting to the variations in the surrounding microenvironment and even to alter it. As a consequence of these alterations, the tumor milieu or microenvironment becomes an “enabling element” for defining some characteristics of cancer cells. For example, the tumor microenvironment can induce cancer cells in acquiring anoikis resistance and in selecting new sites to colonize and grow. Sometimes these cells remain unresponsive until signals generated from the ECM reach the cell's nucleus and they determine whether the cell would proceed to the next stage in cancer progression or not. This response of cancer cells to ECM-generated signals similar to the “dynamic reciprocity” proposed by Bisell for normal cells. An example of such an adaptation of cancer cells to their microenvironment and the resultant clonal selection of invasive cells has been recently reported [4–6].

 Metastatic invasion is generally the final phase of cancer progression, and it involves formation of new blood vessels either by neovasculogenesis, in which endothelial cell precursors (angioblasts) migrate to the tumor site and differentiate and assemble into primitive blood vessels, or by angiogenesis in which we observe sprouting of new blood vessels from preexisting ones, or their longitudinal bifurcation, in the tumor [7]. The invasive tumor cells migrate through these newly formed blood vessels to other sites such as lung and liver brain and this leads to the death of tumor-bearing patients or animals as the case may be.

**Figure 1:**
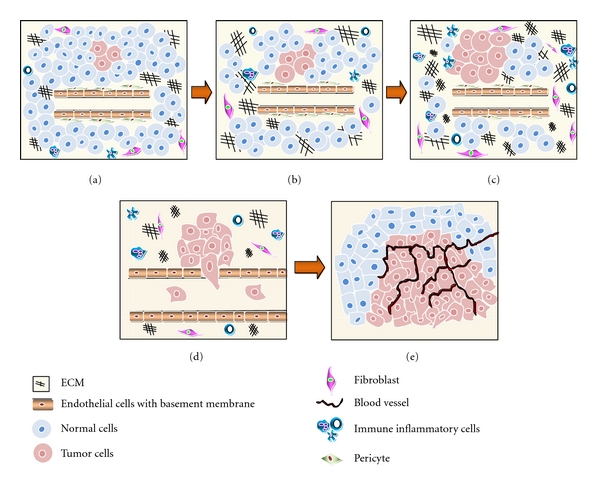
Various steps in tumor initiation and progression where panel (a) represents initiation of tumor by transforming normal cells, panel (b) shows the modulation of ECM proteins allowing transformed cells to multiply, panel (c) shows progression of cancer by replacing normal cells, panel (d) represents the invasion, where cancer cells migrate into the blood stream by modulating ECM and cell adhesion molecules, and panels (e) shows metastasis where the cancer cells are localized at different sites enabling angiogenesis.

Based on available evidence, the entire process of cancer formation can be divided into four different stages: initiation, progression, epithelial mesenchymal transition (EMT), and metastasis (see Figure 1). At the initiation stage, a normal cell acquires oncogenic properties mainly through genetic alterations, which lead to changes in cell structure, adhesion properties, and response to signals from ECM proteins. In the second stage, transformed cells respond to cues from the altered environmental conditions and acquire properties of adhesion-independent growth and colonization. The third stage is also referred to as a transitional or the EMT stage, and, in this stage, the fully transformed cells begin to exhibit mesenchymal gene expression patterns which induce them to invade into the neighbouring tissue and enter into blood circulation [8]. The fourth and prominent stage is metastasis in which the invasive mesenchymes like cells move from the primary site and colonize in a new location. This stage spreads the disease into different parts of the body and involves several alterations in the adhesion properties of cells.

From all earlier observations, it is clear that the cell adhesion in transformed cells plays an important role in all four stages of cancer formation. This paper highlights recent studies done on the integrin-mediated interaction of transformed cells with the ECM and discusses its role in cancer progression.

## 2. ECM Components and Properties

Over the past two decades, research in the field of cancer biology has focussed extensively on the role of ECM constituents during cancer progression. These molecules comprise the cell's microenvironment, and they can affect the mechanical and biophysical properties of cells as well as that of the ECM such as its mechanics, geometry, and topology [9].

In some tissues, mainly of epithelial origin, ECM constituents are present in the basement membrane that defines the boundaries of that tissue. In this location, the organization of these components is different than in the matrix. In the basement membrane, we notice molecules such as collagens, proteoglycans, laminins, and fibronectins associate strongly with certain carbohydrate polymers and generate a membrane-like structure which facilitates the formation of a framework of cells and ECM constituents [10]. Specific domains in ECM proteins that are created by partial gene duplication and exon shuffling during the process of evolution [11] play a critical role in keeping the cells attached to the ECM and the basement membrane and initiating signalling cascades in the cells.

Inside the cells, the ECM-induced signaling pathways are transmitted mainly through integrin molecules that are transmembrane multifunctional ECM receptors. Integrin-mediated signaling in association with many cofactors, for example, cytokines, growth factors, and intracellular adapter molecules, can significantly affect diverse cell processes such as cell cycle progression, migration, and differentiation. The interplay between the biophysical properties of the cell and ECM establishes a dynamic, mechanical reciprocity between the cell and the ECM in which the cell's ability to exert contractile stresses against the extracellular environment balances the elastic resistance of the ECM to that deformation [4, 9]. The ECM in association with the available growth factors activates a sequence of reactions with a complex network of proteases, sulfatases, and possibly other enzymes to liberate and activate various signalling pathways in a highly specific and localized fashion. The maintenance of ECM homeostasis therefore involves a tight balance between biosynthesis of ECM proteins, their 3D organization, cross-linking, and degradation.

## 3. Modulation of ECM-Generated Signaling in Cancer

During cancer progression, we observe significant changes in the structural and mechanical properties of ECM constituents. It has been reported that changes in matrix stiffness, which offers resistance to cell traction forces [12] and also influences “shape dependence” in cells, can contribute actively to the tumor formation [13]. Deregulation of cell shape and alterations in the interactions with the ECM are considered as important hallmarks of cancer cells. These changes in ECM homeostasis can be brought about by the properties of tumor cells themselves or by the secretions of other surrounding cells such as fibroblasts, macrophages, and leukocytes [14]. Integrin ECM interactions are significantly modulated by crosstalk with several other signal-generating molecules, some of them are receptor molecules on the cell surface whereas others are present in the cytoplasm as adaptor proteins and actin-binding proteins [15]. These signaling crosstalks in which integrin molecules lie at the center are very useful for the transition of transformed cells to metastatic cells [16]. Integrin-generated ECM remodelling is further controlled by the localization and activity of proteases [17]. One example of such an integrin-directed cancer progression is seen in breast cancers, where adhesion-independent mammary epithelial cells secrete laminin-5 and luminal cells secrete laminin-1. This leads to aberrant polarity in cells, causing upregulation of metalloproteinases (MMPs, such as MMP9) and induction of tumor invasion and metastasis [18–20].

## 4. Integrins: Its Ligands and Signalling

Integrins are heterodimeric cell-surface receptors that mediate adhesion to ECM and immunoglobulin superfamily molecules. At least 24 distinct integrin heterodimers are formed by the combination of 18 *α*-subunits and 8 *β*-subunits. Specific integrin heterodimers preferentially bind to distinct ECM proteins like laminin, collagen IV, fibronectin, and so forth. The level of integrin expression on the cell surface dictates the efficiency of cell adhesion and migration on different matrices. While some integrins selectively recognise primarily a single ECM protein ligand (e.g., *α*5*β*1 recognises primarily fibronectin), others can bind several ligands (e.g., integrin *α*v*β*3 binds vitronectin, fibronectin, fibrinogen, denatured or proteolysed collagen, and other matrix proteins). Several integrins recognise the tripeptide Arg-Gly-Asp (e.g., *α*v*β*3, *α*5*β*1, *α*IIb*β*3), whereas others recognise alternative short peptide sequences (e.g., integrin *α*4*β*1 recognises EILDV and REDV in alternatively spliced CS-1 fibronectin). Inhibitors of integrin function include function-blocking monoclonal antibodies, peptide antagonists, and small molecule peptide mimetics matrix [21–23].

The positioning of integrin receptors acts as a direct bridge between the extracellular matrix and the internal cell cytoskeleton by transducing key intracellular signals by associating with the clusters of kinases and adaptor proteins in focal adhesion complexes. Integrins thus act as mediators in transmitting different signals from “inside out” (intracellular to extracellular) and “outside in” (extracellular to intracellular) between ECM to cells and vice versa. Through these pathways, ECM proteins are able to control proapoptotic and antiapoptotic cascades by regulating the activity of caspase 8 and caspase 3 [24–26]. ECM-integrin interactions thus determine the balance of apoptotic and cell survival signals and maintain the homeostasis of organs and tissues. Although integrins lack kinase activity, by inter- and intramolecular clustering, they recruit and activate kinases, such as focal adhesion kinases (FAKs) and src family kinases (sFKs) to a focal adhesion complex. In addition to scaffolding molecules, such as p130 CRK-associated substrate (p130CAs; also known as BCAR1), integrins also couple the ECM to actin cytoskeleton by recruiting cytoskeletal proteins, including talin, paxillin, *α*-actinin, tensin, and vinculin. Additionally, they form a ternary complex consisting of an integrin-linked kinase, PINCH, and parvin to regulate many scaffolding and signalling functions required for integrin-mediated effects on cell migration and survival [27].

## 5. Integrin Expression and Signalling in Cancer Progression

Although anchorage-independent growth is a hallmark of malignant transformation, integrins expression levels and activity are an important role in different steps of tumour progression including initiation [28]. Higher expression of *α*3, *α*5, *α*6, *α*v, *β*1, *β*4, *α*6*β*4, *α*9*β*1, *α*v*β*5, and *α*v*β*3 integrins is directly correlated with the progression of the disease [10]. Several epithelial cell tumors showed the altered *α*6*β*4, *α*6*β*1, *α*v*β*5, *α*2*β*1, and *α*3*β*1 integrin expression [29]. Integrin recruitment to membrane microdomains has been shown to be regulated by tetraspanins and crucially regulate integrin function in tumour cells [30]. Recent studies have demonstrated that cell signalling generated by growth factors and oncogenes in transformed cells requires collaboration with specific integrins, especially during tumour initiation. In tumor cells, several survival signals are upregulated upon integrin ligation, which includes increased expression of BCl-2 or FlIP (also known as CFlAR), activation of the PI3K-AKT pathway or nuclear factor-*κ*B (nF-*κ*B) signaling, and/or p53 inactivation [24].

Invasive cancer cells evacuate from the primary site and migrate to the secondary site by the process of tissue invasion and cell migration. Integrin-mediated pathways involving focal adhesion kinase (FAK) and src family kinase (SFK) signaling play a major role in this. In order to survive in the new location and to withstand the stressful conditions of hypoxia, nutrient deprivation, and inflammatory mediators the migratory cells increase the blood supply to themselves by neoangiogenesis. This is achieved by increased expression of *α*v*β*3 and *α*v*β*5 integrins and by deposition of provisional matrix proteins such as vitronectin, fibrinogen, von Willebrand factor, osteopontin, and fibronectin in the tumour microenvironment. Interaction between these molecules plays a critical role in the process of generating new blood vessels in the newly formed tumor site [31, 32].

Integrins found on tumour-associated normal cells, such as the vascular endothelium, perivascular cells, fibroblasts, bone-marrow-derived cells, and platelets, also have a profound effect in tumor progression via integrin-mediated pathways. A summary of these has been given in Table 1 [33–47].

## 6. Genetic and Chromosomal Aberrations at the Onset of Cancer

Neoplasia occurs when cells are exposed to cancer-promoting substances that cause single or multiple premalignant genetic/epigenetic changes which may coalesce to form a large lesion. These genetic changes may have neutral, deleterious, or advantageous effects on the proliferation of a clone or clones of cells. Neutral or deleterious genetic changes may result in stagnation or cell death, whereas the cell receiving advantageous events may result in higher proliferation, recruitment of blood vessels to the developing tumors, and gain the ability to metastasize [48]. The model of Braakhuis et al., 2004 [49], advanced this idea by suggesting that initial genetic alterations occur in stem cells, forming a patch and expanding field of cells with the original and subsequent genomic and or chromosomal alterations. Then, clonal selection of one or more cells within this field of preneoplastic cells leads to the development of a carcinoma. There is a considerable cytogenetic variability among cells reflecting heterogeneity due to clonal evolution within the original tumor [50]. Initial heterogeneity or cell-to-cell differences in cancer are due to cytoskeletal alterations which result in defecting chromosomal segregation and lead to karyotypic variations during mitosis, causing chromosomal aberrations, for example, NUMA1 gene at 11q13, which results in multipolar spindles, leading to daughter cells that differ from each other and their mother cells [51]. Structural chromosome alterations also occur due to deletions, translocations, isochromosomes, dicentric chromosomes, and endoduplicated chromosomes. The gain or amplification of chromosomal segments is driven by more than one gene [52]. Structural rearrangements involving the cleavage and fusion of centromeres from participating chromosomes, also referred to as Robertsonian translocations, are the most frequently observed alterations. Chromosomal aberrations identified with the help of cytogenetic methods including FISH, cCGH, or aCGH showed the gain of the entire long arm of chromosome 3 which amplifies the EGFR gene in SCCHN [53], 8q24 gain to amplify MYC and PTK2 in primary tumors, 11q13 amplifications to amplify cyclin D1 gene, loss of 3q14 causes deletion of fragile site FRA3B/FHIT, necessary to protect cells from accumulation of DNA damage [48]. Aberrations mainly in chromosome 13 and also involving chromosomes 6, 11, 12, and 17 are associated with B-CLL [54].

## 7. Anchorage-Independent Tumor Model System

The wide range of *in vivo* tumor models like syngeneic, human tumor xenograft, orthotopic, metastatic, and genetically engineered mouse models is available from the basis of the compounds selected and treatments that go into clinical testing of patients [55]. The ability to exhibit anchorage-independent cell growth (colony-forming capacity in semisolid media) has been considered to be fundamental in cancer biology because it has been connected with tumor cell aggressiveness *in vivo* such as tumorigenic and metastatic potentials and also utilized as a marker for *in vitro* transformation. Although multiple genetic factors for anchorage-independence have been identified, the molecular basis for this capacity is still largely unknown [56, 57]. During the process of *in vitro* tumorigenesis, various oncogenes with distinct pathways have been shown to transform anchorage-dependent cells to anchorage-independent cells [5, 57]. For example, transfer of c-Myc (a transcription factor), v-Src (a tyrosine kinase), or H-Ras (a small GTPase) into spontaneously immortalized mouse embryonic fibroblasts (MEFs) provides the cells an ability to grow in an anchorage-independent manner [56, 57]. Anchorage-independent multicellular spheroids made by Ewing tumor cell lines were more closely related to primary tumors with respect to cell morphology, cell-cell junctions, proliferative index, and kinase activation [58].

However, changes in ECM and cell adhesion molecular interaction and genetic variations were observed till the date only with the primary or secondary tumors. We have developed a cellular model system by using normal, adherent rat fibroblast cell lines. These cells lose their cytoskeletal organization and specificity to fibronectin as *α*5*β*1 integrins are constantly recycled between cytoplasm and plasma. In the drastic unfavorable stressful conditions, the mechanical, phenotypic, and genetic characteristics are altered/modified to sustain their identity [5, 26]. We observed that this cellular model system represents a tumor with all characteristics of cancer described by Hanahan and Weinberg 2011 as hallmarks of cancer.

**Figure 2:**
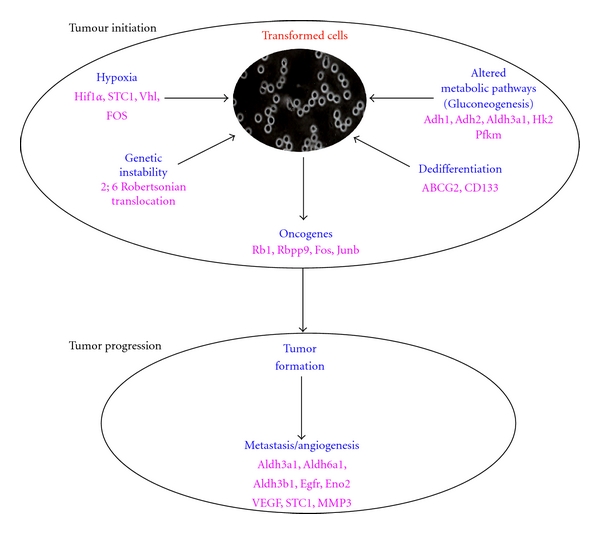
This figure shows the hallmark characteristics of transformed cells at initial stages.

The cells during the nonadhesion process can evade from cell death by caspase 3 interaction with unligated *α*5*β*1 integrins inducing resistance to integrin-mediated death (IMD) and also gain the ability to metastatise. Mutational changes mainly with 2;6 Robertsonian's translocation and activated Ras, FAK, and PKC provide self-sufficient growth signals potential for uncontrollable growth of the cells (Figure 2). Upregulated Spp1, MMP3, Egfr, Rb1, Ddit3, Egln3, Vegfa, Stc1, Hif1a, MMP3, and altered pathways like glycolysis/gluconeogenesis and hypoxia (Pfkm, HK2, Pdk1, Adh1, aldh3a1, and Slc2a) lead the cells to invade, metastasize, and sustain angiogenesis. We observed another phenomenon of dedifferentiation by gaining the stem-cell-like and multidrug resistance properties by expressing Cd133 and ABCG-2 when the cells are exposed to unfavorable condition [5].

The anchorage-independent cellular model system represents a multicentric tumor model system apprehended with genes related to tumor progression, angiogenesis, and metastasis (Figure 2). It is very advantageous, convenient, and possible model system to study the effect of various cancer-mediated drugs at the initial stage itself for the proper diagnosis.

## 8. Integrin Signalling as a Target in Cancer Treatment

Several studies showed the correlation of integrin inhibition at any point of its action will lead to the inhibition of tumor progression [24]. Therefore, integrins are focused pharmacologically in the treatment and prevention of cancer. Antagonists of these integrins suppress cell migration and invasion of primary and transformed cells by inducing apoptosis in primary cells could block tumor angiogenesis and metastasis. Recycling integrins present on the surface of endothelial cells are targeted in the blood stream by exposing to the circulating drugs and agents [59]. Various antibodies, cyclic peptides, disintegrins, and peptidomimetics are meant to bind the targeted integrins to prevent integrin ligation. cRGD, cyclic arginine-glycine-aspartic acid; RGDK, arginine-glycine-aspartic acid-lysine; TRAIL, tumour necrosis factor-related apoptosis-inducing ligand are being used as antagonists integrins to hit the integrin ligand function. The function of upregulated *α*v*β*3 integrin can be blocked by function-blocking monoclonal antibodies, such as LM 609 [60]. The human *α*v integrin specific monoclonal antibody CnTo 95, which targets both *α*v*β*3 and *α*v*β*5 integrins to induce endothelial apoptosis, also had antitumour and antiangiogenic effects in xenograft tumour models [15].

Cilengitide, inhibitor of both *α*v*β*3 and *α*v*β*5 integrins and volociximab, a function-blocking monoclonal antibody against integrin *α*5*β*1, inhibits angiogenesis and impedes tumour growth [61, 62]. *Α*v*β*3 is targeted by various therapeutic antibodies like LM609, vitaxin, humanized mouse monoclonal derived from LM609, CNTO 95, humanized IgG1, c7E3, chimeric mouse human, 17E6, mouse monoclonal antibodies to inhibit tumour growth, and angiogenesis in tumour xenografts and preclinical studies [63]. The prognostic *α*5*β*1 integrins in ovarian cancer can be targeted effectively both *in vitro* and *in vivo* by using specific antibodies. Cell-mediated *α*5*β*1 adhesion can be blocked with a small molecule antagonist (SJ479), a fibronectin-derived peptides in prostate, and colon cancer models [64, 65]. Recent activity is extended to detect tumors and angiogenesis and deliver the drugs to the site of cancer by coupling integrin antagonists to a paramagnetic contrast agent or radionuclide in rabbit and mouse tumour models [66, 67].

## 9. Conclusion

Normal cells lead to the transformation when exposed to adverse conditions such as anchorage independence and effects are found to be similar to the effect of carcinogens and mutagens. These cells could alter the ECM and other cell adhesion molecules by showing altered integrins expression on their surface and are associated with different kinds of growth factors and oncogene. ECM-integrin interactions along with other growth factors provide the diversified anchorage-independent signals to the transformed cells to progress as cancers, metastasis, and angiogenesis. Several tumours are sustained with diversified integrins found to be specific to the tumour-host microenvironment. In future, these integrins can be targeted at the initial stages of cancer by using integrin antagonists to minimize the growth of tumour and metastasis.

## Figures and Tables

**Table 1:** Various integrins in association with different ligands to induce different signaling pathways in generation of tumor and metastasis.

Integrin type	Interacting ECM protein	Activated signaling cascade	Tumor/metastasis	Reference
*α*3*β*1	Laminin	MMP9 and oncogenic Ras, VEGF, FAK-paxillin signaling cascade	Invasion in keratinocytes, Induces angiogenesis, Human hepatoma cells	[33, 34]
*α*6*β*1	Laminin	Urokinase plasminogen activator and MMP-2, PI3Kinase, Src	Tumor invasion in pancreatic cells	[35, 36]
*α*7*β*1	Laminin	Rho-A signaling cascade	Invasion in breast cancer	[37]
*α*2*β*1, *α*1*β*1, *α*10*β*1, *α*II*β*1	Collagen	FAK and src signaling	Invasion of melanoma cells, cancer progression, and invasion of lung adenocarcinoma	[38, 39]
*α*v*β*1, *α*v*β*6, *α*v*β*3	Vitronectin, syndican, thromospondin-1	MMP9, urokinase signaling, MEK/Erk/NF-*κ*B, PKCa, FAK	Metastatic breast Cancer, pancreatic, cervical, colon, lung/liver metastasis	[40–42]
*α*9*β*1	CCN3, osteopontin	Src, P130 Cas, Rac, NOS signaling	Metastatic potential	[43]
*α*IIb3, *α*vb3	Von Willebrand factor	Interacts with thrombospondin-1 and induces VEGF/FGF signaling	Breast cancer	[44, 45]
*α*5	Fibronectin	FAK, ERK, PI-3 K, ILK, and nuclear factor-kappa B -	Metastatic lung and cervical cancer	[46, 47]
*α*L*β*2	Intercellular cell adhesion molecules		Breast cancer	[1]
